# Young People with Intellectual Disability Transitioning to Adulthood: Do Behaviour Trajectories Differ in Those with and without Down Syndrome?

**DOI:** 10.1371/journal.pone.0157667

**Published:** 2016-07-08

**Authors:** Kitty-Rose Foley, John Taffe, Jenny Bourke, Stewart L. Einfeld, Bruce J. Tonge, Julian Trollor, Helen Leonard

**Affiliations:** 1 Department of Developmental Disability, Neuropsychiatry, School of Psychiatry, UNSW Australia, Sydney, Australia; 2 Centre for Developmental Psychiatry and Psychology, School of Psychology, Psychiatry and Psychological Medicine, Monash University, Melbourne, Australia; 3 Telethon Kids Institute, The University of Western Australia, Perth, Australia; 4 Faculty of Health Sciences, University of Sydney, Sydney, Australia; 5 Brain and Mind Research Institute, University of Sydney, Sydney, Australia; University of Tuebingen Medical School, GERMANY

## Abstract

**Background:**

Young people with intellectual disability exhibit substantial and persistent problem behaviours compared with their non-disabled peers. The aim of this study was to compare changes in emotional and behavioural problems for young people with intellectual disability with and without Down syndrome as they transition into adulthood in two different Australian cohorts.

**Methods:**

Emotional and behavioural problems were measured over three time points using the Developmental Behaviour Checklist (DBC) for those with Down syndrome (n = 323 at wave one) and compared to those with intellectual disability of another cause (n = 466 at wave one). Outcome scores were modelled using random effects regression as linear functions of age, Down syndrome status, ability to speak and gender.

**Results:**

DBC scores of those with Down syndrome were lower than those of people without Down syndrome indicating fewer behavioural problems on all scales except communication disturbance. For both groups disruptive, communication disturbance, anxiety and self-absorbed DBC subscales all declined on average over time. There were two important differences between changes in behaviours for these two cohorts. Depressive symptoms did not significantly decline for those with Down syndrome compared to those without Down syndrome. The trajectory of the social relating behaviours subscale differed between these two cohorts, where those with Down syndrome remained relatively steady and, for those with intellectual disability from another cause, the behaviours increased over time.

**Conclusions:**

These results have implications for needed supports and opportunities for engagement in society to buffer against these emotional and behavioural challenges.

## Introduction

Young people with intellectual disability exhibit substantial and persistent problem behaviours compared with their non-disabled peers with a prevalence of psychiatric disorder or major behavioural disturbance of 40% being reported in a population-based cohort of young people with intellectual disability [1]. Problem behaviours in people with intellectual disability have been associated with poorer parental mental health, family quality of life and less likelihood of gaining and retaining employment [2–5]. It has been suggested that these challenging behaviours occur as a result of exposure to a variety of biological, developmental, psychological and social stressors [6, 7]. How the pattern of emotional and behavioural problems changes over time is not well understood.

Down syndrome is the most common known cause of intellectual disability and occurs in 1 in 650 to 1000 live births [8–10]. Affected children experience many medical comorbidities including respiratory and gastrointestinal problems, cardiac and thyroid impairments and vision and hearing deficits [11]. Emotional and behavioural problems have been reported to occur less commonly in Down syndrome than in people with other causes of intellectual disability yet still up to twice as often as in the general population [12, 13]. Estimates of prevalence of depression in adults with Down syndrome vary and range from 0 to approximately 11% [14–16]. For those with intellectual disability without Down syndrome the range of estimates for depression is even wider with reports of between 1% and 39% of the population being affected [17, 18]. As people with Down syndrome enter adolescence, declines in externalising behaviours such as oppositional behaviour and inattention have been observed [19, 20]. During this time, there is a reported increase in internalising behaviours including withdrawal, being more secretive and quiet and preferring to be alone [21]. However differences in the trajectories of domains of psychopathology between young people with intellectual disability with and without Down syndrome have been given scant attention in research. In order to develop optimal and more specific interventions further research is needed to explore the trajectories of these behaviours.

In Australia a longitudinal database of families of young people with Down syndrome from Western Australia [2, 22], found that those young adults who remained in open employment for two consecutive years were significantly more likely, than those in other settings, to experience a decrease in problem behaviours in terms of range, severity and overall behaviour problems [23]. This study highlighted the potential impact of environmental factors on the mental health status of young people with Down syndrome and also emphasized the usefulness of collecting data at various time points.

Another Australian longitudinal database representative of children and young people with intellectual disability, the Australian Child to Adult Development (ACAD) study,[1, 24–26] found the prevalence of psychopathology decreased more in males than in females over time and more in those with mild intellectual disability compared to more severe intellectual disability [1]. A recent report from the ACAD data investigated the association between age and behavioural and emotional problems in healthy ageing adults (up to the age of 56 years) with Down syndrome [27]. Those whose family reported that they had previously received a diagnosis of dementia from a professional, were not included in the study. Once adjusted for age, level of intellectual disability, gender, and medical conditions, no changes in behaviours over time were identified. The authors suggested that they had identified a more positive pattern of ageing in Down syndrome in comparison to previous reports of elevated depressive symptoms [28]. Yet they also highlighted that other studies which identified a decline in externalising behaviours may have had greater sensitivity to subtle age differences and may not have excluded those with dementia [19]. These discrepant findings support and reinforce the importance of considering this group separately from those with intellectual disability of other unknown cause [27].

Understanding different trajectories of psychopathology for different aetiologies of intellectual disability is important for a number of reasons. The decoding of the human genome has, and continues to result in new knowledge of the genetic underpinnings of intellectual disability. This increases the importance of adopting an etiological perspective to research in order to seek and compare different behavioural phenotypes [5, 29]. Additionally research into the increased prevalence of dementia in older people with Down syndrome and its link with a history of depression [30], provides evidence of the importance of gaining a clear understanding of the trajectory of behaviour change over time for these individuals. Identifying specific differences in changes in depressive symptoms, and other elements of psychopathology, may provide markers of increased risk of developing dementia. The earlier that these differences are identified, the better likelihood of successful intervention and treatment.

How behaviour changes over time for people with Down syndrome in comparison to other people with intellectual disability is clearly not yet well understood. A preliminary clinical study also comparing those with Down syndrome versus other intellectual disability found, in contrast to previous literature, those with Down syndrome had significantly higher rates of psychosis (not otherwise specified) or depression with psychotic features[31]. Therefore the aim of this study was to compare changes in emotional and behavioural problems for young people with intellectual disability with and without Down syndrome as they transition into adulthood in two different Australian cohorts.

## Methods

**Fig 1:**
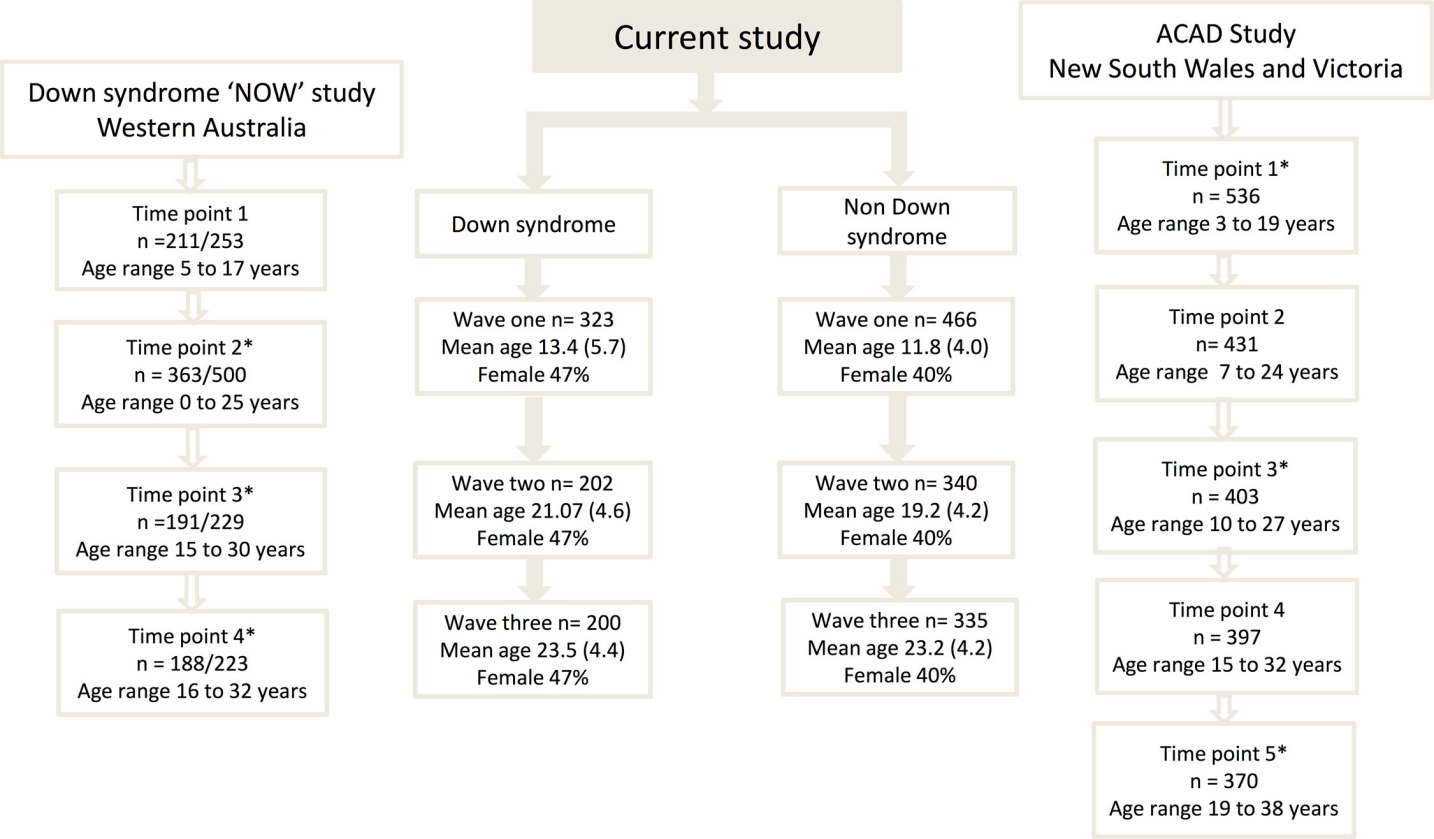
Data collection time points from Down syndrome NOW study, ACAD and the current study

This study examines data collected from three states across Australia using two different databases: the Western Australian Down syndrome ‘Needs Opinion Wishes’ Study and the Australian Child to Adult Development (ACAD) study based across New South Wales and Victoria. We pooled the adults with Down syndrome from all the three states and compared them with adults with intellectual disability with another cause (other intellectual disability) from New South Wales and Victoria (see Fig 1).

### Down syndrome ‘Needs Opinions Wishes’ (NOW) Database

This is a population-based database of young people with Down syndrome residing in Western Australia. The four waves of data collection have occurred in 1997, 2004, 2009 and 2011. Data collection occurred through questionnaires administered to families via paper, over the internet or via telephone interviews. Questionnaires for the last three waves contained two parts pertaining to young person characteristics such as age, gender, emotional and behavioural problems and functioning in activities of daily living. Part two addressed family functioning including family quality of life, communication, and informal and formal supports.

The first time-point of data collection occurred in 1997 with 253 families with a school-aged child with Down syndrome (birth years 1980 to 1991) receiving questionnaires, however data from this wave were not included in the current study as the relevant behavioural outcome measure was not part of this questionnaire.

Time point two, collected in 2004 involved mailing paper copies of questionnaires to families of young people with Down syndrome aged 0 to 25 years (birth years 1980 to 2004), with an option of responding online. Five hundred families received the questionnaires of whom 363 (73%) responded, with 62 having been administered the short version of the questionnaire which did not include the measure of emotional and behavioural problems. Of the 301, sufficient data on 255 individuals (84.7%) were available for this current study. A larger cohort was invited to participate in this wave of data collection in comparison to wave one because this study was not restricted to school-aged children and had a broader focus with inclusion of children and young people under and over school age.

Time points three and four, undertaken in 2009 and 2011 focused on transition from school. Therefore in 2009 questionnaires were distributed to families of 229 young people with Down syndrome aged 15 to 30 years (birth years 1980 to 1994) with 191 (83.4%) families returning questionnaires. Then in 2011 questionnaires were administered to families of 223 young people aged 16 to 32 years (birth years 1980 to 1995) with 188 (84.3%) returning questionnaires. In the comparison of responders and non-responders from the 2004 cohort who were of an appropriate age to participate in subsequent questionnaires in 2009 and 2011, there were no significant differences in any of the six subscales of behaviour. There were no differences in age and gender between responders and non-responders. Participation was consistently high throughout the study due to strategic and consistent participant follow-up.

Consent was inferred if the parents and/or caregivers of the young adults with Down syndrome completed and returned questionnaires. Ethical approval for the Western Australian study was obtained from the Ethics Committee of the Women’s and Children’s Health Services in Western Australia.

### Australian Child to Adult Development Study (ACAD)

ACAD is a representative cohort of children and young people initially sampled from health, education, and family agencies across six census districts across New South Wales and Victoria in Australia. There have been five waves of data collection since 1991. Wave one included 536 children and young people aged 3 to 19 years with intellectual disability. Again participation was consistently high throughout the study with response fractions of 82.5% at wave two, 78.5% at wave three, and 84.0% at wave four, excluding the 31 participants who died since wave one. The heterogeneous aetiology of intellectual disability is reflected in this cohort with diagnostic categorisations being chromosomal or other genetic cause (29%), environmental causes such as prenatal infections, encephalopathy and injury (16%), associated diagnoses such as epilepsy, cerebral palsy, autism and hydrocephalus (28%), and unknown cause (28%). The main attrition in the study was between Waves 1 and 2. There was no significant difference in gender between those lost and not lost to follow-up. Whilst there was no difference in age for the non-Down syndrome part of the cohort, those lost to follow-up in the Down syndrome group were slightly younger (15.3 years for those not lost to follow up, 10.6 years for those lost to follow up, p < .001). Further information on this cohort can be found in previous publications [1, 24, 32].

For the purpose of this study, data from time points 1 (1991–1992), 3 (1999) and 4 (2002–2003) were combined with data from the Down syndrome Needs Opinions Wishes (NOW) study. These time points were selected as the age distributions were similar to those of time points two, three and four of the Down syndrome NOW study (see Fig 1).

All participants in the ACAD study were presented with information and consent forms. If the participants were capable, they signed the forms independently. If the participants were not able to sign the forms, legal guardians consented on their behalf. Institutional review board and ethics approval was obtained for ACAD from the Monash University Standing Committee on Ethics in Research on Humans, Melbourne, Australia; South Eastern Sydney Area Health Service Research Ethics Committee—Eastern Section, Randwick, Australia; and the University of New South Wales Committee on Experimental Procedures Involving Human Subjects, Kensington, Australia.

### Outcome Measure

There are six outcome scores from the Developmental Behaviour Checklist (DBC), an instrument for measuring emotional and behavioural disturbance from a parent or carers perspective, developed specifically for children and young people with intellectual and/or developmental disability [33]. The DBC-A has high test-retest and inter-rater reliability ranging from 0.72 to 0.85 and satisfactory concurrent validity with two measures of emotional and behavioural disturbance (32). The measure’s total score has been reported to be strongly associated with child psychiatrists’ ratings of psychopathology (R = 0.81, P < .001) and to be sensitive to change [1, 34]. In both the ACAD and the Down syndrome NOW study, the parent/carer report DBC was employed to measure psychopathology.

In this study the scores were presented as mean item scores (MIS) of each of the subscales of the DBC. The subscales described disruptive (e.g. abusive, swears, tells lies, stubborn and disobedient), communication/disturbance (e.g. talks to self or imaginary people, repeats back what others say), anxiety (e.g. distressed about being alone, fears particular things or situations, upset or distressed over small changes), social relating (e.g. doesn’t show affection, aloof, in his/her own world, resists being cuddled or touched), depression (e.g. unhappy, confused, withdrawn, lost enjoyment) and self-absorbed behaviours (e.g. hums, whines, groans, bangs head, eats non-food items).

There are two versions of the DBC, the primary carer version (DBC-P) for use with children (4–18 years) and the adolescent/adult version (DBC-A; 19 years plus)[33, 35]. The DBC-P is made up of 96-items and the DBC-A 107 items. Responses are scored on a three point scale 0 “Not true as far as you know” 1 “Somewhat or sometimes true” and 2 “Very true or often true.” [36]. Five of the subscales were based on the factors of the DBC-P and the sixth based on the depression scale. Where the DBC-A was used, the subscales were calculated based on the same set of items as the DBC-P. In respect to the depression scale, the score was the mean of the list of DBC-P depression items if the DBC-P was used and the mean of the DBC-A factor subscale if the DBC-A was used. The DBC-A depressive symptoms subscale has been found to have good validity and reliability for young people in the adolescent/adult age ranges and therefore applicable to the specific waves of data collection with participants of this age [33]. The DBC-P was used for wave one of the Down syndrome NOW study and waves one, two and three of the ACAD study. There are minor differences between the DBC-P and DBC-A reflecting the need for slightly different questions for adolescents and adults compared to children in terms of emotional and behavioural disturbance.

Communication skills were measured in both the ACAD and the Down syndrome NOW study. A question asking whether the young person was ‘somewhat’ or ‘able to speak’ versus the young person being ‘unable’ to use speech to communicate was available and the variable was converted to binary in both cohorts in order to compare behaviour changes in those who were ‘able’ or ‘somewhat able’ to speak compared to those ‘unable’ to speak.

### Data Analysis

This current study includes three waves from each study which will be referred to as wave one, two and three in chronological order.

Outcome scores were modelled using random effects regression as linear functions of age, Down syndrome status, ability to speak and gender. In these analyses age was decomposed into two components, average age during the study, which varies only between people, and individuals’ deviations from their averages, which vary only within people. The deviations from average age during the study are the measure of time under observation, and coefficients of this variable are estimates of average rates of change per year in the outcomes. The effect of age in Down syndrome and non-Down syndrome was also presented for each subscale.

## Results

Participants in the Down syndrome ‘NOW’ database (n = 255) were combined with those from the ACAD study who had Down syndrome (n = 68). These young people’s behaviour (n = 323) was compared to those young people who had intellectual disability of another cause from the ACAD study (n = 466). Summary statistics of age, gender and ability to speak for each group are shown (Table 1).

**Table 1:** Summary statistics of age, proportion female and proportion able to speak in the Down syndrome and non-Down syndrome samples at the 3 data waves, with sample sizes.

		Intellectual disability/ Non Down syndrome	Down syndrome
Wave 1	Frequency	466	323
	Mean age in years (SD)	11.78 (4.02)	13.42 (5.70)
	Female (proportion)	.40	.47
	Able to speak (proportion)	.75	.71
Wave 2	Frequency	340	202
	Mean age in years (SD)	19.20 (4.16)	20.96 (4.57)
	Female (proportion)	.40	.48
	Able to speak (proportion)	.75	.77
Wave 3	Frequency	335	200
	Mean age in years (SD)	23.23 (4.19)	23.54 (4.44)
	Female (proportion)	.40	.47
	Able to speak (proportion)	.75	.77

*Note*. SD, standard deviation.

**Fig 2:**
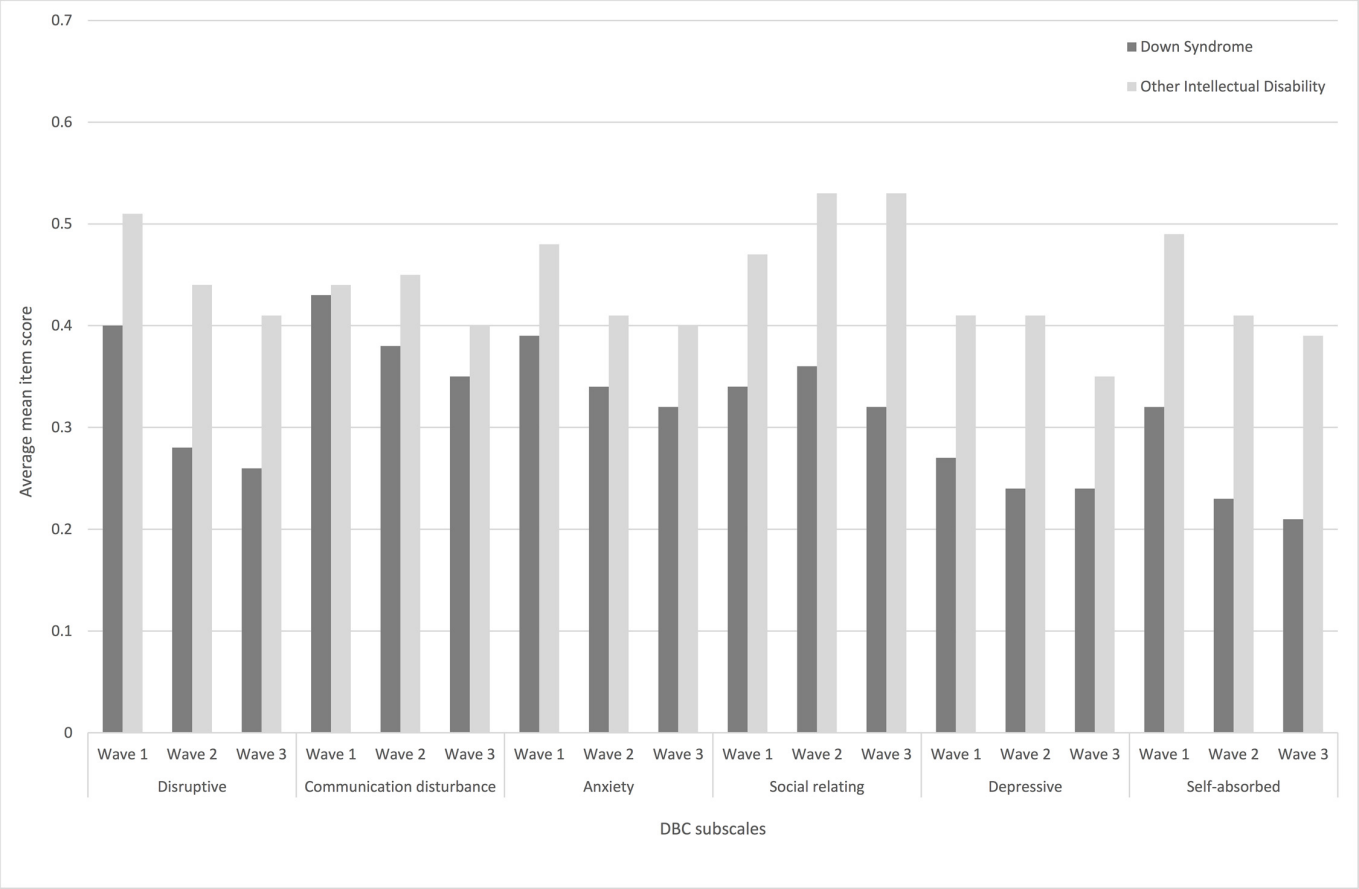
Average levels of mean item scores on six behaviour subscales of the DBC in those with Down syndrome and other intellectual disability over three waves of data collection

Subscales describing disruptive, self-absorbed and anxious behaviours were lower at each consecutive time point for both those with Down syndrome and intellectual disability of another cause (Fig 2). Except for the social-relating subscale all scales showed a decrease from wave one to wave three for both groups. The social-relating subscale increased (ie there were more problems) over time for those with intellectual disability of another cause but not for Down syndrome.

### Regression Analysis

In a longitudinal study age varies in two ways. It differs between participants and it changes within participants as they are observed in the study. A focus of this study is to investigate whether change in response variables with ageing (trajectories) differ on average between groups based on source of ID. The first variable in column 1 of Table 2, a person’s *average age* during the study, reflects age differences between people. The second variable, *ageing*, is based on the person’s differences from his/her own average age at the different time points of the study. We fit an interaction (the fourth variable) between ageing and group (non-Down syndrome, Down syndrome) to allow estimation of whether possible ageing effects differ between these groups. The *ageing* coefficient estimates the ageing effect for the non-Down syndrome group, and the addition of the *ageing* and the ageing*Down syndrome coefficients is the estimate of the ageing effect for the Down syndrome group. A significant ageing*Down syndrome coefficient is evidence that the Down syndrome group trajectories differ on average from the non-Down syndrome group trajectories.

**Table 2:** Regressions of six DBC subscales on age, Down syndrome status, ability to speak and gender.

	Regression coefficients (effect size)					
	Disruptive	Communication/disturbance	Anxiety	Social relating	Depressive symptoms	Self-absorbed
average age in study	-.007 (.12)‡	-.005 (.09)†	-.008 (.14)‡	.003 (.05)	.001 (.02)	-.007 (.14)‡
ageing in study¥	-.010 (.13)‡	-.004 (.05)‡	-.007 (.09)‡	.008 (.10) ‡	-.002 (.03)	-.010 (.15)‡
Down syndrome ˠ	-.106 (.33)‡	-.040 (.13)	-.069 (.21)‡	-.163 (.48)‡	-.122 (.38)‡	-.166 (.60)‡
Ageing*Down syndrome	-.005 (.05)	-.005 (.05)	.003 (.03)	-.008 (.08)‡	-.001 (.01)	.002 (.02)
Speech	.070 (.22)‡	.110 (.35)‡	.009 (.03)	-.120 (.35)‡	-.015 (.05)	-.221 (.80)‡
Female gender	-.003 (.01)	-.028 (.09)	.033 (.10)	.016 (.05)	.045 (.14)‡	-.023 (.08)
Number of observations	1682	1683	1682	1683	1682	1683
Number of individuals	655	655	655	655	655	655

Note A higher score indicates worse behaviour; age coefficients reflect per year changes.

ˠ Reference is those with intellectual disability without Down syndrome.

¥ Within person deviations from mean age in study

†p<0.05

‡p<0.01.

After possible age-related effects are accounted for, the variables Down syndrome (0 non-Down syndrome, 1 Down syndrome), speech (0 not able to speak, 1 able to speak) and female gender (0 male, 1 female) account for averaged-across-time-points differences between the responses in these pairs of groups.

Time trajectories of the DBC disruptive, communication, anxiety and self-absorbed subscales showed similar patterns of decline (interpreted as improvement, since higher DBC scores indicate more problematic behaviour) as the participants age, in a similar way for both Down syndrome and non-Down syndrome groups (significant *ageing* coefficients combined with non-significant ageing*Down syndrome coefficients). The depressive subscale did not change significantly with ageing in either group, and the social relating subscale increased with ageing (at .008 units on the 0–2 scale per year) for the non-Down syndrome group but did not change over time in the Down syndrome group (significant -.008 ageing*Down syndrome coefficient cancels the *ageing* coefficient).

After trajectories are accounted for, the average scores for participants with Down syndrome are considerably lower on all 6 DBC subscales than those of participants without Down syndrome.

On average across groups based on source of ID and over time, ability to speak is positively associated with the disruptive and communication subscales (indicating poorer behaviour) and negatively associated with the social relating and self absorbed subscales (indicating better behaviour), all with sizeable effects. Female gender is positively associated with the depressive subscale, with a small effect (indicating poorer behaviour).

## Discussion

This study investigated change in behaviour over time for people with intellectual disability and the effect of cause of intellectual disability, being Down syndrome or other cause. Behaviour generally improved over time for all individuals with intellectual disability, a finding mirrored in other studies [13]. However we found two important differences between changes in subscales of behaviours between those with Down syndrome and those with another cause of their intellectual disability.

Firstly, the depression scores for both those with and without Down syndrome did not change significantly with age, suggesting no improvement in depressive symptoms compared with the other behavioural domains. The lack of a decline in depressive symptoms in our study is of concern especially since symptoms of depression may become even more apparent for people with Down syndrome later in life or in association with onset of dementia [37, 38]. Despite some evidence that individuals with Down syndrome have a specific vulnerability to experiencing depression [14, 39–41], we found that overall they, as in other behavioural domains, exhibited fewer depressive symptoms than those with another cause of intellectual disability. The trajectory of changes over time, was the more concerning element. Our findings highlight the importance of continuing careful assessment of adults with Down syndrome for symptoms of depression, which may be difficult to identify and may be a precursor to dementia[42].

Secondly, abnormal social relating behaviours (e.g. loner, not affectionate, avoids eye contact, sits and watches others) increased for those with other intellectual disabilities over the course of the study (signifying a deterioration in this area of behaviour with age) and remained relatively consistent for those with Down syndrome. It is well reported that children and young people with intellectual disabilities experience difficulties developing social skills and in overall social competence [43, 44]. Adolescence and young adulthood is also a period of significant physical, sexual and emotional development. It is a time when many young adults with intellectual disability become aware of what having a disability means for their lives [45]. A realisation of their disability, difficulties with social skills in the increasingly complex social world of adolescents and adults and hormonal changes during adolescence, may influence social relating behaviours for young people with intellectual disability at this time. The apparently better social relating skills in those with Down syndrome could be due to a number of reasons. Specific strengths in socialisation skills have been reported in teenagers with Down syndrome and the more identifiable facial features of the syndrome result in their intellectual disability being immediately recognizable and therefore acknowledged [45, 46]. Also, researchers have identified a ‘Down syndrome advantage,’ with people with Down syndrome reported as reaching better outcomes than people with other developmental disabilities [5, 47, 48]. People with mild intellectual disability have overall been found to be more likely to be socioeconomically disadvantaged [49, 50]. Those with Down syndrome, whose socio-economic status is more representative of the general population may be comparatively more advantaged, and have more family supports leading to a better overall self-esteem. These factors could be playing a role in the differences we found in the trajectories of social relating behaviours between those with Down syndrome and those with other intellectual disability. For those with other intellectual disabilities, the higher social relating scores may also reflect greater discrepancy of social skills from the raters’ or parents’ hopes or expectations. Poorer social relating behaviours have also been associated with increased maternal depression and anxiety for families with young people with intellectual disability [26].

Young people with intellectual disability who were able to speak exhibited more communication disturbance problems, depressive symptoms and disruptive behaviours but less self-absorbed and social relating problems. It may be assumed that individuals who were unable to communicate by speech were lower functioning with a more severe intellectual disability. However, this result could also be an artefact of the measure. Additionally, our findings could be highlighting the difficulties in identifying symptoms of depression in people with intellectual disabilities who are unable to communicate by speech, rather than showing that those who were able to speak experienced more depressive symptoms. This needs to be further examined before conclusions are drawn.

There were some limitations in the study. Measuring emotional and behavioural difficulties in young people with intellectual disability presents challenges when the young people themselves are not able to self-report. In this study, the majority of the data were parent-report. Research in the general population has suggested that parental and self-reporting of emotional and behavioural problems differs, specifically in regards to internalising behaviours. [51] Identifying reliable methods and measures for direct reporting from individuals with intellectual disability is an important area for future research [52, 53]. However, the use of the psychometrically rigorous and valid measure, the DBC, provides a widely used, reliable carer-report method of measuring emotional and behavioural disturbances for this population [54]. A further limitation was the wide range of ages included in our populations. Although we adjusted for age in the statistical analysis, the impact of this limitation should always be considered when interpreting the results. This study involved data from two different cohorts from three different states, Western Australia, New South Wales and Victoria. Differences in state-based policies and services could have influenced behavioural outcomes for the young people in this study. Information on ethnicity and sociodemographic status was not available for both cohorts and there may have been some differences in these factors between the groups.

The different and more persistent trajectories of the depression and social relating subscales of behaviour we observed have specific practical implications. Health professionals treating people with intellectual disability can use this knowledge to guide their service, in terms of specific behaviours to assess and target for behavioural interventions. Individuals with intellectual disability also need to be facilitated to engage in the social community to ensure they sustain rich and varied activities for cognitive stimulation and skill development. Continued community and occupational engagement may buffer against the onset of depression or the decline in social relating behaviours [55].

## Conclusion

This study has found that people with Down syndrome experience less behavioural problems than people with intellectual disability of another cause across all subscales of emotional and behavioural problems, except for communication disturbance. Depressive symptoms did not significantly decline for those with Down syndrome compared to those without Down syndrome. The trajectory of the social relating behaviours subscale differed between these two cohorts, where those with Down syndrome remained relatively steady and, for those with intellectual disability of another cause, the behaviours increased over time. The findings from this study provide valuable information for health and other professionals working with people with intellectual disability.
